# Smartphone-Based Passive Sensing of Activity Levels and Behavioral Activation During Psychosocial Interventions for Older Adults With Depression: Longitudinal Observational Study

**DOI:** 10.2196/92348

**Published:** 2026-09-30

**Authors:** Soohyun Kim, Oded Bein, Emily Carter, Hongzhe Zhang, Jihui L Diaz, Zilong Yu, Patricia Marino, Jo Anne Sirey, Dimitris Kiosses, George Alexopoulos, Nili Solomonov, Samprit Banerjee

**Affiliations:** 1 Department of Population Health Sciences, Weill Cornell Medicine, 425 E 61st St, Dv319, New York, NY, 10065-6304, United States, 1 347-267-6424; 2 Department of Psychiatry, Weill Cornell Medicine, New York, NY, United States; 3 Perelman School of Medicine, University of Pennsylvania, Philadelphia, PA, United States; 4 School of Nursing, Duke University, Durham, NC, United States

**Keywords:** mHealth, mobile health, passive sensing, behavioral activation, late-life depression, functional data analysis

## Abstract

**Background:**

A key symptom of depression is reduced behavioral activation, namely, low activity levels and reduced meaningful engagement with the external environment. Thus, objective and timely measures of activity levels are useful tools to precisely track individuals’ activity levels during treatment. Prior adult depression studies have shown that activity levels measured using passive sensing (eg, step counts and time spent away from home) predict depression relapse, persistence, and poor response to psychosocial interventions. However, there is scarce research on how passive sensing measures relate to behavioral activation, especially in late-life depression.

**Objective:**

This study aimed to examine the association between passive sensing activity levels and self-reported behavioral activation during psychosocial interventions in community-dwelling older adults with depression.

**Methods:**

The sample comprised depressed older adults from 3 clinical trials at the Weill Cornell ALACRITY Center (N=75; mean age 70.7, SD 8.34 y; n=68, 90.7% female; n=46, 61.3% White). Participants were randomized to 9 weeks of either behavioral interventions or comparison conditions. Activity levels were measured by smartphone-recorded daily step count and time away from home. Self-reported behavioral activation was measured using the BADS (Behavioral Activation for Depression Scale). We applied a functional regression model (scalar-on-function) to test the association between activity levels and pre-post intervention changes in behavioral activation.

**Results:**

In the psychotherapy group, higher step count showed a positive pointwise association with greater improvement in behavioral activation during the final phase of treatment (days 46‐63). At the start of this interval, the estimated coefficient was β(46)=1.51 (95% CI 0.07-2.94), increasing to β(63)=6.61 (95% CI 0.80-12.41). In the active control group, greater time away from home showed a negative pointwise association with behavioral activation from day 2 to day 49. The estimate was β(2)=−0.04 (95% CI −0.07 to −0.001) and β(49)=−0.50 (95% CI −0.98 to −0.03) at day 49, with the largest negative estimate at day 32 (β[32]=−0.75, 95% CI −1.37 to −0.13). These pointwise intervals are descriptive and not multiplicity-adjusted.

**Conclusions:**

These preliminary, hypothesis-generating findings suggest that passive sensing in older adults can measure changes in activity levels during interventions for late-life depression. Because clinical change was modest and between-group functional differences were not formally tested, passive sensing should be interpreted as a complementary, low-burden measure rather than a replacement for validated clinical assessments.

## Introduction

Reduced behavioral activation—manifested as low activity levels and diminished engagement with the external environment—is a core symptom and treatment target in late-life depression. Accordingly, objective and timely measures of activity levels (eg, walking, distance traveled) are essential to inform interventions for late-life depression. The activity levels of individuals predict depression relapse, persistence, and poor response to psychosocial interventions [1-3]. Historically, studies of activity levels have relied predominantly on self-report measures. Self-reports are typically collected only at periodic intervals, missing the daily dynamics and even moment-to-moment fluctuations in behavioral patterns [4,5].

At the same time, validated self-report instruments of psychological constructs, such as behavioral activation, ask about the subjective, goal-directed quality of engagement, and are psychometrically reliable. The goal of passive sensing is, therefore, not to replace such assessments, but to evaluate whether continuously collected sensor-derived measures can feasibly and reliably provide complementary information about behavioral activation during treatment. This is especially important because behavioral change during depression treatment may be nonlinear, with clinically meaningful fluctuations occurring between scheduled assessment visits [6,7]. Passive sensing measures, collected without user input via sensors on mobile health (mHealth) devices (eg, smartphones and wearables), are a promising complement, providing continuous and unobtrusive assessments of daily behaviors potentially linked to mental health. These measures provide objective, granular activity data that can potentially reveal behavioral patterns associated with therapeutic response [8]. These data can, therefore, be used to advance the development and refinement of interventions for older adults with depression.

This study focused on examining whether activity levels, measured with simple and cost-effective smartphone-based passive sensing, are linked with individuals’ self-reported behavioral activation. Late-life depression is characterized by reduced behavioral activation—low activity and engagement [9] with the external environment. Tracking behavioral activation via objective and dynamic passive sensing measures can inform early risk detection and timely interventions. We focused on 2 inferences from raw sensor data—step count and time spent away from home—as these behaviors capture core elements of behavioral activation, such as engagement with the external environment. Prior observational studies in adults and older adults [10] showed that increased time spent at home [11-14] and limited physical activity [11,12,14-16] are associated with higher depression severity. Research on late-life depression has been limited, likely due to technological barriers in this age group [17,18] and misperceptions that older adults cannot use technology [19-21]. Our work and that of others have shown the feasibility of using technology to augment treatment in older adults [22]. However, it is currently unknown whether passive sensing correlates with clinical outcomes in this population [23,24].

Passive sensing can be especially beneficial when trajectories of change are nonlinear and require frequent measurements. Prior research has demonstrated nonlinear changes in symptoms and behaviors during treatment, including fluctuations that precede meaningful improvement in symptoms [7,25]. In prior pilot work, we examined passive sensing activity trajectories in a very small cohort of depressed older adults (n=3) and found that visually inspected patterns of passive sensing activity appeared to align with self-reported behavioral activation over time [20]. However, this preliminary work was exploratory and descriptive.

Building on this pilot evidence, the present study examined the association between passive sensing activity levels and self-reported behavioral activation during psychosocial interventions in community-dwelling older adults with depression. Participants were randomized to 9 weeks of either simple psychosocial interventions, based on behavioral activation principles, or a control condition, both delivered by community therapists. We leveraged functional regression, an approach that enables us to detect dynamic and nonlinear patterns in activity over time and their correlations with behavioral activation. We hypothesized that fluctuations in the trajectory of activity levels would correlate with self-reported pre-post changes in behavioral activation. If this hypothesis is supported, the findings would demonstrate the feasibility of using passive sensing data to monitor activity in this population and inform the development of interventions that encourage behavioral activation and enhance treatment response. We use the BADS (Behavioral Activation for Depression Scale) as the clinical referent precisely because it is a validated and reliable instrument: establishing whether densely sampled sensor data track a psychometrically sound measure of behavioral activation is a necessary first step before sensor-derived activity can be interpreted clinically. The aim is, therefore, not to argue that passive sensing should displace clinical measures like the BADS, but to determine whether continuously collected, objective activity indices carry information about the construct the BADS measures. The BADS, however, can only be administered at a small number of visits, and therefore cannot resolve the day-to-day dynamics of behavior during treatment. The contribution of this work is thus to test whether an objective, continuously measured signal is meaningfully linked to a sparse but reliable clinical measure, and to characterize how that link varies across the course of treatment—something neither measure can reveal on its own.

## Methods

### Participants

The sample included 130 participants from 3 clinical trials at the Weill Cornell ALACRITY Center (P50MH113838): Reaching and Engaging Depressed Senior Center Clients (REDS), Improving Depression in Elder Mistreatment Victims (EM/PROTECT), and A Behavioral Intervention for Depression and Chronic Pain in Primary Care (RELIEF). Participants were middle-aged and older adults (≥50 y) with significant depressive symptoms (9-item Patient Health Questionnaire [PHQ-9] score ≥10 [26]). Exclusion criteria included active suicidal ideation (Montgomery-Asberg Depression Rating Scale [MADRS] item 10 score ≥4 [27]) and any diagnosis other than unipolar depression or generalized anxiety disorder (*Diagnostic and Statistical Manual of Mental Disorders*, Fifth Edition [*DSM-5*]) [28], cognitive impairment, or severe or life-threatening medical illness. Of the 130 participants (84 intervention and 46 comparison), 96 (63 intervention and 33 comparison) had both clinical and passive data.

Passive sensing with consumer smartphones differs from data collection with research-grade devices in a key respect: there is no supervised setting in which wear status can be verified. The phone logs data only during periods when the participant happens to have it on their person, and days on which the device was set aside cannot be distinguished from days of true low activity—a pattern that may also shift within the same participant across the study period. If left unaddressed, this ambiguity systematically deflates passively derived measures. We therefore screened each participant-day with the 2SpamH (2-Step Preprocessing Algorithm for mHealth Data) algorithm, which classifies individual days as unreliable or missing due to device nonuse (eg, the phone not being carried); values on flagged days were subsequently imputed. By removing device nonuse days from the data and imputing them, 2SpamH prevents nonwear from being mistaken for inactivity and thereby mitigates the systematic underestimation of activity that would otherwise result. Device nonuse was common but comparable across treatment arms: the mean per-participant proportion of device-observed days classified as nonuse was 0.532 (SD 0.338) for the intervention group vs 0.510 (SD 0.347) in the control group for step count, and 0.489 (SD 0.329) vs 0.486 (SD 0.355) for time at home. Thus, roughly half of device-observed days per participant were flagged and imputed, with no meaningful difference between arms, indicating that 2SpamH filtering was unlikely to introduce differential missingness by treatment group.

Furthermore, participants showing evidence of nonwear, noncompliance, or device malfunction were excluded. Specifically, these cases were characterized by extremely low or flat activity patterns across multiple consecutive days, often accompanied by large segments of missing data and very low total volumes of recorded data (ie, fewer than 21 d with usable nonzero step count data). Flagged participants typically displayed long stretches of identical or near-zero values punctuated by a few (approximately 1‐3) isolated spikes, consistent with intermittent device carriage or synchronization errors rather than true low activity due to depression. After excluding 6 flagged cases and those missing BADS [9] data at baseline or week 9, the final analytic sample included 75 participants (49 treatment and 26 control). Studies were approved by Weill Cornell Medicine’s Institutional Review Board (IRB).

### Trial Registration

The 3 parent trials were registered at ClinicalTrials.gov: REDS I (NCT03246789), EM/PROTECT (NCT03241225), and RELIEF (NCT03265210).

### Ethical Considerations

All 3 parent studies were approved by the Weill Cornell Medicine IRB. The protocol numbers were as follows: REDS I (1704018114), EM/PROTECT (1703018101), and RELIEF (1704018104). All participants provided informed consent before study participation.

### Treatments

Participants were randomized to either (1) a psychosocial intervention based on behavioral principles, delivered by community therapists (licensed social workers and mental health counselors) [7,10], or (2) an active comparison condition. All participants in the 3 trials were middle-aged and older adults with depression, studied in 3 distinct contexts: community-dwelling senior center clients (REDS), older adults who experienced abuse (EM/PROTECT), and adults with co-occurring chronic pain and depression (RELIEF). Critically, all 3 trials delivered psychotherapies for depression that shared the same therapeutic target, behavioral activation, and all 3 used BADS to measure that target. The samples, therefore, differ in the context in which depression was experienced and treated, not in the construct being treated or measured, which supports pooling them to address the present questions. Across all 3 studies, interventions were similar, including behavioral activation as one of the key targets to improve depressive symptoms in these 3 populations. In the REDS study, participants who were clients of a New York City senior center received either a group-based behavioral activation psychotherapy based on *Engage*, delivered in senior centers (intervention), or psychoeducation (wellness of body and mind) and a referral to primary care or a senior center clinic (comparison) [29,30]. In the EM/PROTECT study, participants were older adults who experienced abuse and were referred from agencies serving older adults experiencing abuse in New York City. They received a behavioral activation therapy tailored for older adults experiencing abuse in the community (intervention) or older adult mistreatment services and care as usual (comparison) [31]. In the RELIEF study, participants with chronic pain and depression were recruited from primary care practices in New York City and received a behavioral intervention tailored for chronic pain and depression delivered in primary care settings (intervention) or care as usual (comparison).

### Measures

#### Passive Sensing Activity Measures

We tracked daily step count and time away from home during the 9-week (63 d) study using an mHealth app on a smartphone [32]. The “time away from home” measure was inferred using raw sensor data from GPS and accelerometers, using algorithms described previously [33]. Daily summaries of passive sensing data were stored on a platform developed by HealthRhythms. Because passively sensed data depend on whether participants carry their smartphones, periods of apparent inactivity may reflect either true low activity or device nonuse. To address this ambiguity, we applied our preprocessing pipeline, the 2SpamH algorithm [22], which predicts device nonuse via auxiliary device engagement metrics using an unsupervised 2-stage k-nearest neighbors algorithm. We applied the 2SpamH algorithm to the daily aggregate step count and time away from home measures to infer device nonuse. Days that the algorithm estimated as nonuse were labeled as missing data. Following our prior work [22], we then imputed the missing data using the MissForest algorithm [34], incorporating all behavioral variables collected via the app—not limited to step count and time away from home.

#### Clinical Measures

Trained independent raters, unaware of treatment assignment, conducted study assessments in the participant’s home or a safe public location at baseline, mid-treatment (week 6), and at treatment end (week 9). We measured self-reported behavioral activation using the BADS [9]. The BADS is the gold-standard measure of behavioral activation, which is the primary target of the psychosocial interventions studied here and the mechanism through which these treatments aim to improve depressive symptoms. Although the BADS is self-reported, it is a validated instrument of established psychometric quality and is not the kind of retrospective activity log for which passive sensing is proposed as an alternative. The BADS has shown high reliability and internal consistency in prior works [9,35,36] and in this sample (Cronbach α=0.74; 95% CI 0.64-0.84). Increases in BADS scores over time indicate greater behavioral activation, reflecting improved engagement in goal-directed activity and better response to psychotherapy. The BADS comprises 25 items, each rated from 0 (not at all) to 6 (completely), yielding a total score ranging from 0 to 150, with higher scores indicating greater behavioral activation. A minimal clinically important difference for the BADS total score is approximately 9 to 10 points, derived using the reliable change index, which accounts for the test-retest reliability of the instrument [9]. We selected BADS as the clinical referent for this reason: it is a validated measure of the subjective, goal-directed dimension of activation, which sensor data cannot capture directly. Its limitation in the present context is not measurement error but temporal resolution—it was administered at only 3 visits and therefore cannot characterize day-to-day behavioral dynamics. This motivates our analytic strategy, in which continuously sampled passive sensing trajectories are related to change on a sparse but reliable clinical measure.

#### Statistical Analysis

Means and SDs of demographics and BADS scores were computed and compared between treatment groups using Kruskal-Wallis tests for continuous demographic and clinical variables and Fisher exact tests for categorical variables. Although the primary analyses focused on within-person associations between activity and behavioral activation, baseline comparisons were conducted to confirm that intervention and comparison groups were demographically and clinically similar at study entry, ensuring interpretability of group-level patterns. To examine overall changes in behavioral activation and test for group differences over time, we fit linear mixed-effects models for repeated measures of BADS. The models included fixed effects for time (baseline, week 6, and week 9), treatment group (psychotherapy vs active control), and their interaction (time × group), with random intercepts for participants to account for within-person correlation. These models were specified on observed BADS scores rather than on pre-post change scores. The main effect of time tested whether behavioral activation improved across the study period within each group, whereas the time × group interaction tested whether the magnitude of change differed between the psychotherapy and active control groups (ie, treatment effect). The reported model-adjusted mean changes from baseline to week 9 were subsequently derived as estimated marginal contrasts from the mixed-effects models using the *emmeans* package in R. Accordingly, the change scores reflect contrasts from the repeated-measures mixed-effects models rather than outcomes of a separate change-score regression.

We applied a scalar-on-function regression, which models a scalar outcome (ie, change in BADS score from baseline to week 9 or end of treatment) as a nonlinear function of time-varying predictors (daily summaries of step count and time away from home). This approach allowed us to examine temporally heterogeneous nonlinear associations between pre-post change in behavioral activation (BADS) and passive sensing measures: daily step count (scaled by 1000) and time away from home (hours) across the 9-week psychotherapy period, separately for each treatment group (4 regressions total). This functional regression approach is well-suited for passive sensing data because it (1) captures temporal dependencies, (2) models complex, dynamic, and potentially nonlinear relationships, and (3) reduces dimensionality by representing densely sampled data as smooth functions. For example, increased step count early in the intervention may have a different effect on the pre-post change in BADS scores than later in the intervention. By modeling dense time-dependent measures as smooth functions of time, this approach reduces measurement noise while preserving the temporal structure of the data. Because participants were pooled across 3 parent trials, all functional regression models were adjusted for study membership (REDS, EM/PROTECT, and RELIEF), and the study-adjusted models are reported as the primary analysis. Separate treatment group-specific models were used to describe within-group temporal patterns and to maintain interpretability in this exploratory secondary analysis.

We projected each participant’s passive sensing trajectories (step count and time away from home) onto *K* orthonormal natural-spline cubic basis functions, where *K* denotes the number of basis functions. Cubic splines were chosen as they are the lowest-order polynomials that ensure continuity of the curve and its first 2 derivatives, providing a smooth and stable representation of the data. We considered *K*∈{4,...,8} basis functions and selected *K*=5 by minimizing the Bayesian information criterion [37]; improvements in the Bayesian information criterion for *K*>5 were minimal across all models, indicating that additional basis functions did not meaningfully improve model fit. The same *K* value was retained across models to facilitate comparability of the estimated coefficient functions β(*t*) over time across predictors and treatment groups, rather than allowing model-specific smoothing that could obscure cross-model interpretation. In our analysis, the knot construction generated one internal knot at the midpoint of the study period after removing the first and last points of the equally spaced knot sequence as boundary knots. A natural cubic spline with 1 internal knot and 2 boundary knots spans 2 basis functions. The *K*=5 specification, therefore, yielded a 63 × 2 natural spline basis matrix, with 63 rows corresponding to study days and 2 columns corresponding to the 2 resulting basis functions. Each participant’s activity trajectory was consequently summarized by 2 spline basis coefficients, and the functional predictor contributed 2 *df* to each model. This low-dimensional representation guards against overfitting in our study with a modest sample size. The day-level coefficient function β(*t*) was then reconstructed as a deterministic linear combination of the spline basis functions, $B{\left(t\right)}^{T}\hat{\gamma }$, where *B*(*t*) denotes the natural spline basis evaluated at day *t* and $\hat{\gamma }$ denotes the fitted regression coefficients for the corresponding basis terms. Thus, β(*t*) was not estimated independently at each study day, but was obtained by evaluating the same smooth, low-dimensional coefficient function across the 9-week intervention period.

Pointwise 95% CIs for β(*t*) were computed from the estimated covariance matrix of the fitted basis-coefficient estimates. Specifically, if $\hat{\Sigma }(\hat{\gamma })$ denotes the estimated covariance matrix of $\hat{\gamma }$, then the pointwise variance of $\beta \left(\hat{\gamma }\right)$ was calculated as $Var\left[\hat{\beta }\left(t\right)\right]=Var\left[B{\left(t\right)}^{T}\hat{\gamma }\right]=\;B{\left(t\right)}^{T}\hat{\Sigma }B\left(t\right)$. The corresponding pointwise 95% CI was calculated as $\hat{\beta }\left(t\right)\pm t_{0.975}df\;\times \;SD\left(t\right)$, where $SD\left(t\right)=\sqrt{\{}diag[B\left(t\right)\;\hat{\Sigma }(\hat{\gamma })B(t{)}^{T}]\}$ is the pointwise SD at day *t*, and *df* denotes the residual df from the fitted regression model. This computation uses the full covariance matrix of the basis coefficients, rather than their marginal SEs, and therefore accounts for correlation among the fitted spline-basis coefficients. These intervals are presented as pointwise summaries of uncertainty in the estimated coefficient function and are not interpreted as multiplicity-adjusted evidence of statistically significant day-level effects. These intervals are presented as pointwise summaries of uncertainty in the estimated coefficient function and are not interpreted as multiplicity-adjusted evidence of statistically significant day-level effects.

To aid interpretability of the estimated coefficient function β(*t*) over time (days), the coefficient at day 1 was set to 0. This normalization step removes the arbitrary intercept in the functional regression and allows β(*t*) to represent deviations in the association between activity and behavioral activation relative to the beginning of the intervention. Accordingly, results are described starting from day 2, when the first estimated time-varying effects appear. Note that because of this normalization, variances at early time points adjacent to day 1 are compressed. Each participant’s trajectory was represented as a weighted sum of 2 natural-spline cubic basis functions. This smoothing step supports the 3 advantages of scalar-on-function regression noted above by (1) preserving the temporal structure of behavior, (2) allowing for time-varying effects of activity (eg, step count may have different associations with BADS early vs late in treatment), and (3) filtering day-to-day noise while reducing dimensionality to yield stable predictors. The smoothed curves of participants then served as predictors in scalar-on-function regressions that modeled pre-post change in BADS as an integral over the smoothed curves, estimating a continuous β(*t*) coefficient function over the 9-week intervention. β(*t*) reflects the unique contribution of the activity on day *t* to the overall pre-post change in BADS, after accounting for activity in other days. All analyses were conducted using R statistical software version 4.4.2. Throughout, we describe the estimated coefficient function in terms of whether its 95% confidence band included 0 at a given time point; intervals whose bands included 0 are reported as such and are not interpreted as evidence of a day-level effect.

## Results

### Participant Characteristics

The analysis included 75 older adults (mean age 70.7, SD 8.34 y; n=68, 90.7% female; n=46, 61.3% White; Table 1). Those in the behavioral activation intervention group had more years of education, compared to those in the comparison group (mean 15.4, SD 2.65 vs mean 13.6, SD 3.84; *P*=.04), with no other significant differences (Figure 1; Table 1).

**Table 1.** Demographics and clinical characteristics of the overall sample and subsamples by treatment group (N=75).

Characteristics	Overall	Group		
		Comparison (n=26)	Behavioral activation interventions (n=49)	*P* value^a^
Age (y), mean (SD)	70.7 (8.34)	72.2 (8.17)	70.0 (8.48)	.23
Sex: female, n (%)	68 (90.7)	24 (92.3)	44 (89.8)	>.99
Years of education (y), mean (SD)	14.8 (3.23)	13.6 (3.84)	15.4 (2.65)	.04
Baseline BADS^b^, mean (SD)	89.5 (20.02)	89.0 (22.37)	89.7 (18.90)	.92
Week 9 BADS, mean (SD)	94.7 (24.78)	91.3 (27.57)	96.6 (23.26)	.52
ΔBADS (baseline to wk 9)^c^, model-adjusted mean (SE)	4.59 (2.87)	2.93 (4.66)	6.25 (3.36)	.56^d^
Race, n (%)				.59
White	46 (61.3)	14 (53.8)	32 (65.3)	
Black	22 (29.3)	10 (38.5)	12 (24.5)	
Asian	1 (1.3)	0 (0.0)	1 (2.0)	
Other	6 (8.0)	2 (7.7)	4 (8.2)	
Ethnicity				
Hispanic	10 (13.3)	2 (7.7)	8 (16.3)	.48
Marital status, n (%)				.63
Single	20 (26.7)	6 (23.1)	14 (28.6)	
Married	16 (21.3)	8 (30.8)	8 (16.3)	
Separated	5 (6.7)	1 (3.9)	4 (8.2)	
Divorced	12 (16.0)	3 (11.5)	9 (18.4)	
Widowed	22 (29.3)	8 (30.8)	14 (28.6)	
Study, n (%)				.06
PROTECT	18 (24.0)	10 (38.5)	8 (16.3)	
REDS	28 (37.3)	10 (38.5)	18 (36.7)	
RELIEF	29 (38.7)	6 (23.1)	23 (46.9)	

a *P* values refer to the difference between psychotherapy and active control groups. They were calculated using the Kruskal-Wallis test for continuous variables and Fisher exact test for categorical variables.

b BADS: Behavioral Activation for Depression Scale.

c Values represent model-adjusted contrasts from a repeated-measures linear mixed-effects model fit to observed BADS scores at baseline, week 6, and week 9 using *emmeans*; change scores were not modeled directly.

d *P* value corresponds to the difference-in-differences (psychotherapy and active control groups) in change from baseline to week 9, using the Kenward-Roger method for df. Within-group *P* values were .06 for the psychotherapy group and .52 for the active control group.

**Figure 1.**
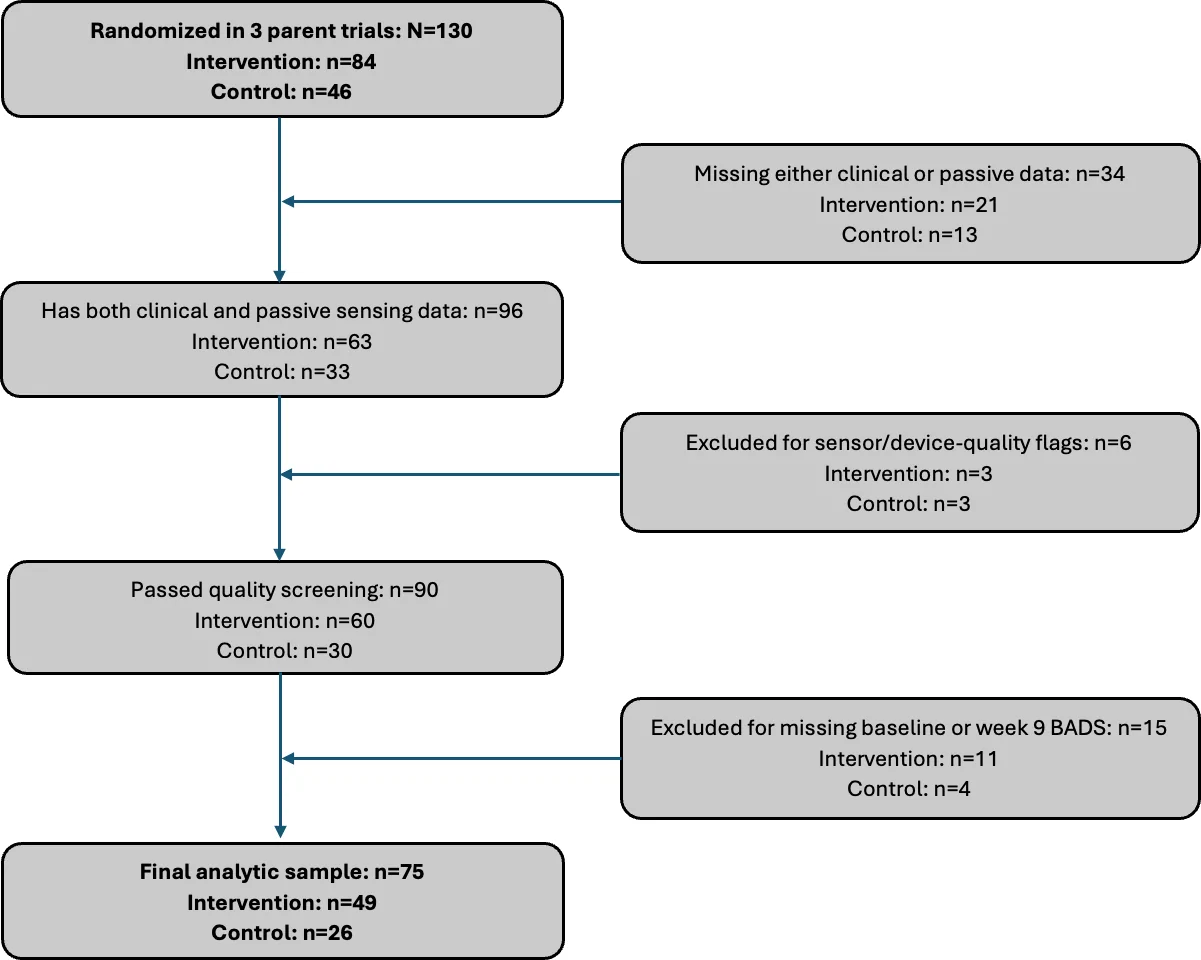
Participant flow from randomized parent-trial sample to final analytic sample. BADS: Behavioral Activation for Depression Scale.

### Behavioral Activation Trajectories in Treatment Groups

Behavioral activation, measured by BADS, was examined longitudinally using linear mixed-effects models fit to repeated assessments at baseline, week 6, and week 9. As shown in Figure 2A, model-adjusted estimated marginal means (EMMs) for the overall sample demonstrated a modest upward trajectory in behavioral activation over the 9-week study period. The model-adjusted EMM change in BADS score from baseline to week 9 was +4.64 points (SE 2.87), for which the 95% confidence band included 0 (95% CI −1.03 to 10.31; *P*=.11). The 95% confidence band for the overall main effect of time also included 0 (*F*_2,144.78_=2.03; *P*=.13).

Group-specific trajectories are displayed in Figure 2B, which illustrates differential patterns of change by treatment condition. Examination of the interaction terms indicated that, relative to the active control group, participants receiving psychotherapy exhibited a larger model-adjusted increase in behavioral activation by week 6 (time × treatment interaction at week 6: estimate=+11.45, SE=5.80; 95% CI −0.02 to 22.91; *P*=.049). This early divergence is visually apparent in Figure 2B, where the psychotherapy group demonstrates a steeper increase in BADS scores at mid-treatment compared to the active control group. However, the additional difference at week 9 was smaller, and its 95% confidence band included 0 (estimate=+3.32, SE=5.74; 95% CI −8.03 to 14.70; *P*=.56). Consistent with these interaction patterns, within-group contrasts showed that participants in the psychotherapy group demonstrated a larger improvement from baseline to week 9 (EMM change=+6.3, SE=3.36; 95% CI −0.35 to 12.95; *P*=.06) with marginal significance (ie, *P*<.10), whereas no significant change was observed in the active control group (EMM change=+2.98, SE=4.66; 95% CI −6.22 to 12.18; *P*=.52).

Overall, these results indicate modest improvements in behavioral activation over time, with evidence of an early divergence in trajectories favoring psychotherapy, which attenuated by the end of treatment, with the 95% confidence band for the between-group difference at week 9 including 0. Given this temporal heterogeneity, we next examined whether passive sensing measures captured time-specific behavioral patterns that were differentially associated with changes in behavioral activation over the course of treatment. Specifically, we evaluated time-varying associations between daily activity patterns (step count and time away from home) and pre-post changes in BADS using scalar-on-function regression.

**Figure 2.**
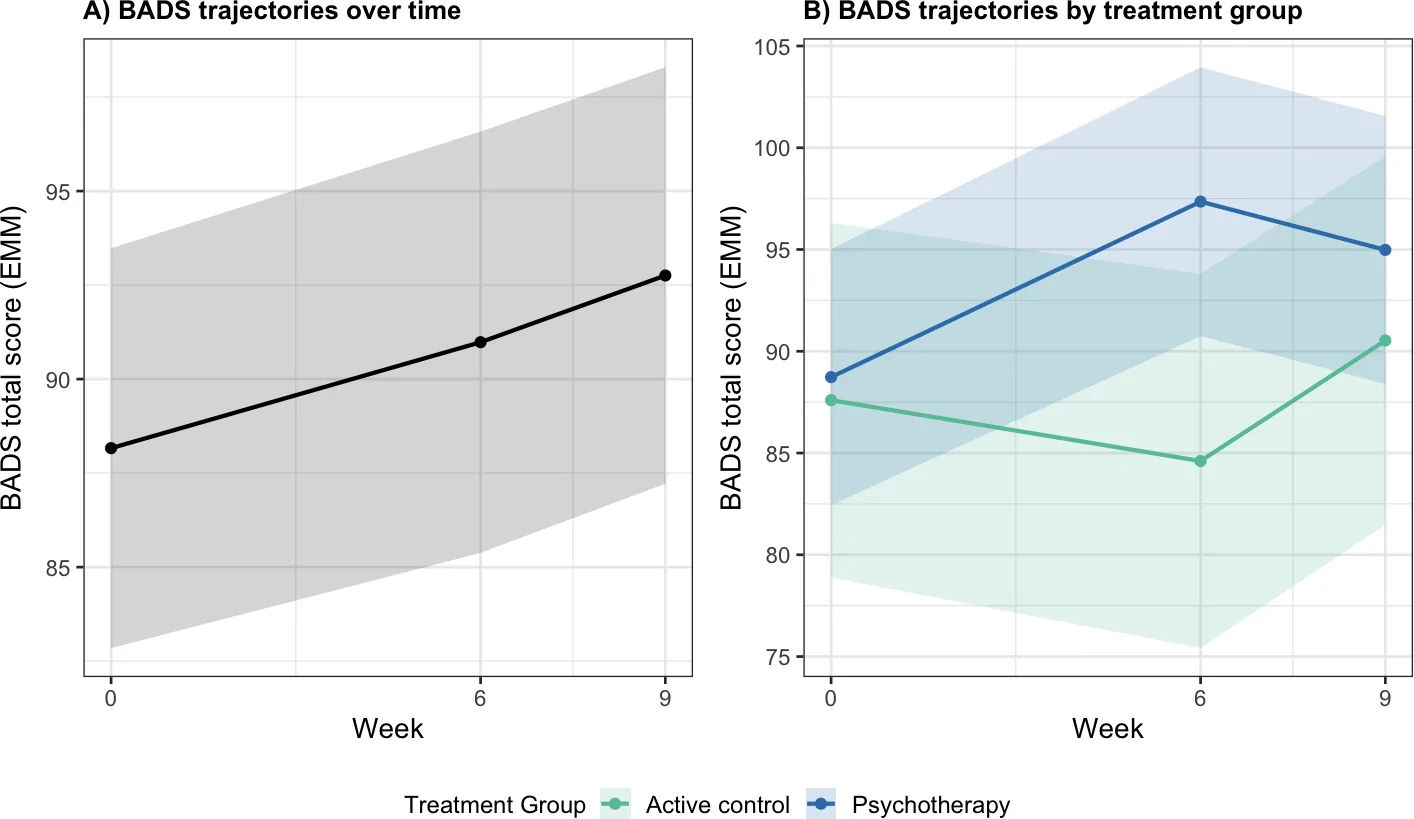
Model-adjusted estimated marginal means (EMMs) of BADS scores across baseline, week 6, and week 9 are shown for the (A) overall sample and (B) by treatment condition. Shaded bands indicate 95% CIs. BADS: Behavioral Activation for Depression Scale.

### Association Between Step Count and Behavioral Activation

Figure 3A illustrates the dynamic association between step count and improvement in behavioral activation (ie, pre-post change in BADS score) over the 9-week study period. In this coefficient profile over time plot, positive β(*t*) values indicate that higher step count is associated with greater improvements in behavioral activation from baseline to week 9 (ie, higher BADS scores, which indicate improvements in BADS), whereas negative β(*t*) values indicate that higher step count is associated with less improvement or declines in behavioral activation from baseline to week 9. In the psychotherapy group, the estimated coefficient curve showed an initially negative association between step count and behavioral activation earlier in treatment, followed by a transition to a positive association later in treatment. Although the early negative portion of the curve did not reach statistical significance, this pattern suggests that the direction of the step count and behavioral activation association changed over the course of treatment. Higher step count was positively associated with greater improvement in behavioral activation during the final phase of treatment, from day 46 through day 63. At the start of this interval, the estimated coefficient was β(46)=1.51 (95% CI 0.07-2.94), and the association strengthened through the end of the intervention, reaching β(63)=6.61 (95% CI 0.80-12.41) at day 63. The active control group did not show intervals in which the pointwise 95% CI for the step count coefficient excluded 0. These intervals are interpreted as descriptive pointwise summaries rather than multiplicity-adjusted tests of day-level significance. Of the 9 weeks, no significant association was detected.

**Figure 3.**
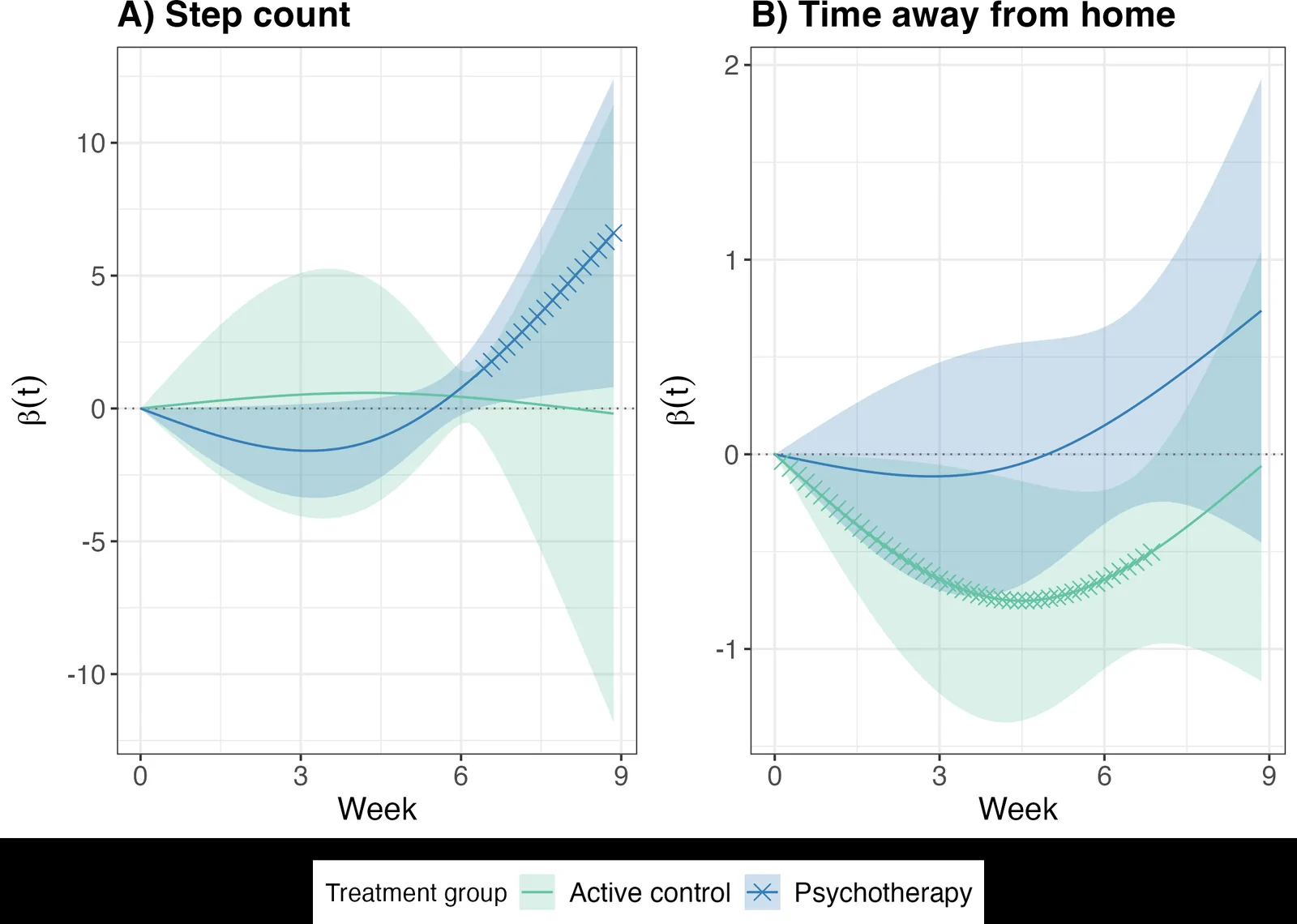
Estimated functional coefficients β(*t*) relating (A) step count and (B) time away from home to pre-post change in BADS during psychotherapy and active control. Shaded bands indicate 95% CIs; tick marks denote time points where β(*t*) differs from 0. The intercept was constrained to β(0)=0.

### Association Between Time Away From Home and Behavioral Activation

Figure 3B illustrates the dynamic association between time away from home and behavioral activation over the 9-week study period. In this coefficient over time plot, positive β(*t*) values indicate that more time spent away from home is associated with greater improvements in behavioral activation (ie, higher BADS scores), whereas negative β(*t*) values indicate more time spent away from home is associated with less improvement or declines in behavioral activation. In the psychotherapy group, the pointwise 95% CI for time away from home did not exclude 0 over a sustained interval. In contrast, the active control group demonstrated a negative pointwise association between time away from home and behavioral activation from day 2 through day 49. At day 2, the estimate was β(2)=−0.04 (95% CI −0.07 to −0.001), and at the end of the interval, day 49, the estimate was β(49)=−0.50 (95% CI −0.98 to −0.03). The largest negative estimate occurred at day 32 (β[32]=−0.75, 95% CI −1.37 to −0.13). This pattern suggests that, in the active control group, spending more time away from home was descriptively associated with less improvement in behavioral activation across the early-to-middle portion of the study period.

## Discussion

Our study found preliminary evidence that fluctuations in smartphone-derived passive sensing activity levels are dynamically associated with changes in behavioral activation among participants undergoing interventions for late-life depression. These findings should be interpreted as hypothesis-generating and descriptive because our analysis describes pointwise associations rather than formally tested between-group differences. In the intervention group, the relationship between activity levels (step count and time away from home) and improvement in behavioral activation changed over time. The estimated coefficient curve was negative earlier in treatment and shifted to a positive association in the final weeks, although the early negative portion of the curve had a 95% confidence band that included 0. Higher step count was positively associated with greater improvement in behavioral activation during the final phase of treatment, from day 46 to day 63. In contrast, the comparison group did not show intervals in which the pointwise 95% CI for the step count coefficient excluded 0. For time away from home, the comparison group showed a negative association with changes in behavioral activation from day 2 to day 49, whereas the intervention group did not show a comparable interval in which the pointwise CI excluded 0. These patterns suggest that passively sensed activity may provide complementary information about behavioral activation during treatment, particularly in the later phase of psychotherapy, but they should not be interpreted as multiplicity-adjusted tests of day-level significance.

The observed shift from an early negative association between activity levels and improvement in self-reported behavioral activation in the initial phase (1‐6 wk) may be explained by the structure of the therapy itself. The interventions begin with a focus on psychoeducation and goal setting in the first few weeks, shifting their focus to techniques to change behavior in the later phase of the intervention [30,31]. It is possible that, as patients begin engaging more with assignments that target behavioral activation later in the intervention, passive activity levels are more strongly and positively associated with improvements in behavioral activation. This interpretation remains speculative and should be evaluated in adequately powered prospective studies with prespecified functional interaction tests.

Another possible explanation for the shift from an early negative to a later positive association between activity levels and self-reported improvements in behavioral activation could be changes in patients’ perceptions of the benefits of treatment during the course of the intervention. Late-life depression is characterized by a negativity bias, which skews attention toward negative rather than positive information and thoughts [30,38,39]. This bias can heighten skepticism toward positive treatment outcomes, reducing engagement with the core therapeutic element—behavioral activation—designed to increase activity levels. As the intervention progresses, patients may become more self-aware of their behaviors and experience a reduction in negativity bias. This increased self-awareness can enhance engagement with the core therapeutic element. In contrast, the consistent negative association in the control group may reflect persistent negativity bias and low self-awareness of behavioral patterns, explaining the negative association. These factors can lead patients to report low behavioral activation even when they are more active.

We adopted functional regression, a methodology that enables smoothed analysis of nonlinear dynamic associations between activity levels and behavioral activation. This approach revealed nonlinear-temporal associations between passively sensed activity levels and behavioral activation, highlighting the importance of models that can capture such patterns [40]. Functional regression transforms raw, noisy passive sensing activity data into smoothed trajectories, allowing us to examine gradual changes in the association of activity levels with behavioral activation over time in a way that is clinically interpretable. This methodology simplifies densely sampled and noisy passive digital data, extracting interpretable and dynamic trends. To the best of our knowledge, our study is the first to apply functional regression to passive sensing in late-life depression to examine the relationship between objective passive measures and subjective self-report symptom measures [41,42].

Our study has several limitations. First, we were unable to provide an overall statistical test of association comparing the intervention and control conditions because 2-group statistical tests for functional regression have not yet been developed. We report pointwise estimates of association with CIs for both groups over time. Future work should use a single scalar-on-function model with a prespecified group × activity functional interaction or a permutation-based global test of between-group differences. Second, we combined samples from 3 studies of behavioral activation-based psychotherapies to enhance statistical power, which limited our ability to examine population-specific trajectories in the 3 studies. Pooling was supported by the fact that all 3 trials treated depression in middle-aged and older adults and shared behavioral activation, measured by BADS, as the therapeutic target; the 3 samples differed in the context of depression rather than in the treated construct. We adjusted all functional models for study membership, and descriptive patterns were largely unchanged between adjusted and unadjusted models, but the sample remains too small to estimate population-specific trajectories within each of the 3 studies. Third, we note that a positive relationship between passive sensing and behavioral activation can also emerge when both measures decrease together. Our ability to examine this was limited because behavioral activation was not measured as frequently as passive sensing. Therefore, we examined how the dynamics of activity levels during interventions correlated with pre-post changes in behavioral activation. Future research could record behavioral activation daily and examine how passive sensing activity levels and self-reported behavioral activation track daily. However, such daily self-reporting dramatically increases patient burden, making it less feasible in clinical research and practice. This underscores the importance of real-time, consistent, and passive monitoring to capture changes during interventions.

In conclusion, the results demonstrate the feasibility of measuring activity levels with passive sensing during interventions for late-life depression. Our findings suggest that passive sensing activity levels are associated with self-reported behavioral activation in later phases of brief interventions for late-life depression. Because sensor-derived activity tracks a validated measure of behavioral activation, it offers a continuous, low-burden complement to periodic clinical assessment. We emphasize that passive sensing is not proposed as a substitute for validated instruments such as BADS, which capture the subjective and goal-directed quality of engagement that sensors cannot observe. Rather, it may reduce reliance on retrospective self-reported logs of behavior itself [8,18]. The application of methods that capture nonlinear trends, such as functional regression, is optimal for addressing the fluctuations and noise in passive sensing data. Therefore, our study serves as a motivation to track passively sensed activity levels during behavioral activation–based psychotherapies, as increases in activity levels serve as an indicator of increased behavioral activation, particularly in the later stages of the therapy [18,43].

## Abbreviations

2SpamH: 2-Step Preprocessing Algorithm for mHealth Data
BADS: Behavioral Activation for Depression Scale
*DSM-5*: *Diagnostic and Statistical Manual of Mental Disorders*, Fifth Edition
EM/PROTECT: Improving Depression in Elder Mistreatment Victims
EMM: estimated marginal mean
IRB: institutional review board
MADRS: Montgomery-Asberg Depression Rating Scale
mHealth: mobile health
PHQ-9: 9-item Patient Health Questionnaire
REDS: Reaching and Engaging Depressed Senior Center Clients
RELIEF: A Behavioral Intervention for Depression and Chronic Pain in Primary Care
